# Perceptions of alcohol use disorder support among liver transplant recipients: a survey of strategies and challenges

**DOI:** 10.3389/frtra.2025.1577086

**Published:** 2025-06-26

**Authors:** Maria P. Cote, Natalia Rodríguez-Payan, Srilakshmi Atthota, Nahel Elias, Leigh Anne Dageforde

**Affiliations:** ^1^ Department of Surgery, Massachusetts General Hospital, Boston, MA, United States; ^2^ Department of Surgery, Maimonides Medical Center, Brooklyn, NY, United States; ^3^ Department of Surgery, Harvard Medical School, Boston, MA, United States

**Keywords:** alcohol use disorder, return to alcohol use, liver transplant, patient perceptions, resources

## Abstract

**Background:**

Alcohol use disorder (AUD) treatment in liver transplant (LT) recipients requires multidisciplinary management. We aim to analyze post-LT patients' perceptions of the transplant clinic, local community resources, desired supports and barriers for AUD recovery resources.

**Methods:**

A survey of adult recipients who received a LT within the last ten years with a history of AUD at a single Transplant Center was conducted. The survey consisted of five categories: demographics, strategies for AUD treatment used before and after LT, recent alcohol use, and challenges faced in AUD treatment. Results were reported using descriptive statistics.

**Results:**

Forty-one of 203 approached participants completed the questionnaire over a 3-month period [median age 56 years (45.5–62), 68.3% male, 90.2% white, median time since transplant 21 months (9.4–50.7)]. Thirty-three (80.5%) had a period of abstinence from alcohol prior to LT: 17 (41.5%) 1–5 years, 7 (17%) 6–12 months, and 7 (17%) < 6 months. 88.9% reported their goal for alcohol was complete abstinence. Useful strategies for AUD management before LT included exercise (73.1%), family support (63.4%), and therapy (58.5%). Exercise was most effective post-LT resource to prevent return to alcohol use, followed by social work assistance (51.2%), and finding a new hobby (48.8%). Social support and difficulties with availability of AUD treatment resources were the main challenges perceived by survey participants.

**Conclusion:**

Exercise, social support, social work assistance, finding new hobbies, and therapy were the preferred resources for AUD management. Future interventions should facilitate access to resources to assist with sobriety and incorporate their outside support network in assisting with recovery from AUD.

## Introduction

Alcohol related liver disease is a leading indication for liver transplant (LT) in the United States (1). Notably, up to 22% of post-transplant patients return to alcohol use at one year after LT and 40% of patients are using alcohol by 5 years. This post-LT alcohol use is associated with increased mortality rates due to recurrent cirrhosis and rejection when patients stop their immunosuppression medications (2, 3). Despite this rate of alcohol use after LT potentially leading to graft loss and death, there is no widely implemented standardized care model for liver transplant patients with alcohol use disorder (AUD) (4).

Risk factors for return to alcohol use after LT have been identified, including psychiatric comorbidities, particularly post-transplant depression, smoking, younger age, and family history of AUD (5). Managing AUD after LT requires comprehensive care coordination and a multidisciplinary approach with an integrated team of transplant specialists, addiction specialists, and mental health experts (4, 6). Our previous work studied providers perceptions of challenges to appropriate post -LT AUD care. Providers cited patients' ongoing desire to drink and denial of alcohol misuse as some factors limiting post-LT AUD recovery (7). Providers also identified lack of post-transplant support for patients, insufficient mental health professionals in the post-LT space, and needing specific staff to address AUD treatment post-LT (7).

While there are multiple different treatment strategies for AUD, and some knowledge of provider perspectives on the management options, little is known about what post-LT patients perceive to be most useful. In addition, post-LT patients may not be aware of available resources and/or may have difficulty accessing these resources. This lack of knowledge can limit the appropriate resource allocation to optimize AUD care in the post-LT setting. While other studies have assessed patient perceptions of resources to support AUD management in the setting of alcohol associated liver disease (8), To our knowledge, there are no other studies that focus on patients' perspectives on the availability and usefulness of resources for AUD management after LT. We aim to describe and analyze the perception of known resources available to post-LT patients and the challenges patients encounter in AUD recovery after LT.

## Methods

This study was deemed exempt by the Massachusetts General Hospital Institutional Review Board under protocol number 2023P001432. Given the minimal risk for participants, signedformal signed consent was waived. Completion of the survey served as consent.

### Participant selection

Adult (>21 years old) liver transplant recipients with a history of alcohol use disorder in the setting of alcohol associated liver disease and a liver transplant within the last 10 years who had previously signed consent to be contacted for research were invited to participate. All patients included were >21 years old at the time of transplant and survey completion.

### Subject recruitment

Patients who met the inclusion criteria and had previously signed consent for research were approached via phone call or in-person at the time of their transplant center clinic visit and were offered information on the study and a study fact sheet. Patients who were contacted on the phone with an upcoming appointment were asked if they were willing to complete the survey in person at the time of their transplant clinic visit. Patients who did not have an upcoming visit were contacted via phone, given the fact sheet, and if agreeable to participation were emailed a link to complete the survey online. Completion of the survey was considered consent for participation.

### Instrument development and administration

A 28-item survey was developed to assess the challenges perceived by patients about AUD management after transplant. The survey was modified for patients from a survey previously administered to transplant providers. The provider survey had been developed after review of the literature in peri-LT AUD by experts in transplant psychology and psychiatry, addictions, hepatology, and surgery. It was then pilot tested prior to being administered nationally to transplant professionals (7).

The adapted patient survey was divided into five sections as follows (Supplement 1): 1. Demographic questions (age, self-reported gender and race/ethnicity), 2. resources used before liver transplant (yes/no to 17 different resources), 3. resources used after liver transplant (yes/no to 16 different resources), 4. challenges in receiving adequate AUD care after LT, including social support, availability of community resources, logistic challenges, and liver transplant-specific challenges (yes/no to 25 challenges across 4 elements), and 5. experience with alcohol use, including sobriety prior to transplant (yes/no and duration of this period), current goal for alcohol use from patients and perceived goal of the transplant center, and the validated AUDIT-C and Stages of Change questionnaires (9, 10). For this survey, sobriety was defined as no use of alcohol. All sections had an open-ended option for participants to respond with additional elements they considered important to mention different from the resources stated.

The survey then underwent review by a multidisciplinary research team and was pilot tested to assess for any survey design flaw, understanding of the questions, and the estimated time requirement for completion for respondents.

### Data collection

The survey was administered from March to September 2024 using REDCap, a secure web-based survey administration tool (11). Variables on previous and current psychiatric comorbidities were collected from electronic health records of participants and recorded in the same REDCap. Partial responses from participants were included in the analysis. Respondents who completed the entire survey were given a US $10.00 e-Gift card as renumeration.

### Statistical analysis

Statistical analysis was performed using Stata 17.0 (StataCorp, College Station, Texas). Categorical variables were reported as frequencies and percentages, and continuous variables were reported as median and interquartile range. For open-ended questions, text responses were reviewed and grouped based on frequency regarding the challenges and available resources for AUD care described by participants.

## Results

### Participant characteristics and experience with alcohol use

A total of 203 patients were contacted either in-person or via phone call, of which 41 agreed and completed the survey, with a response rate of 20.2%. Thirty-nine completed the questionnaire fully, but the partial responses from two were still included in the study results. The median age of respondents was 56 years (45.5–62). The majority were white (92%), and 68.3% were male, with a median time since transplant of 21 months (9.43–50.7). 43.9% of participants presented with psychiatric comorbidities in addition to alcohol use disorder, of which 29.3% had more than one comorbidity. The most common psychiatric comorbidity was major depressive disorder (29.3%) followed by generalized anxiety disorder (26.8%) (Table 1).

**Table 1:** Demographic characteristics of patient population.

Variable	Median (IQR) or *N* (%)
Age	56 (45.5–62)
Self-reported gender	
Female	13 (31.7)
Male	28 (68.3)
Other	0
Self-reported race/ethnicity	
White	37 (90.2)
Black	2 (4.9)
Asian	1 (2.4)
Hispanic/Latino	1 (2.4)
Time since transplant	639 (287–1,542)
Presence of psychiatric comorbidities	
Yes	18 (43.9)
No	23 (56.1)
Presence of more than 1 psychiatric comorbidity	12 (29.3)
Psychiatric comorbidities	
Major depressive disorder	12 (29.3)
Bipolar disorder	2 (4.9)
Generalized anxiety disorder	11 (26.8)
Post-traumatic stress disorder (PTSD)	2 (4.9)
Attention deficit and hyperactive disorder (ADHD)	3 (7.3)
Substance abuse disorder (different from alcohol)	2 (4.9)
Other	2 (4.9)
Period of abstinence from alcohol before transplantation	
Yes	33 (80.5)
No	3 (7.3)
No answer	5 (12.2)
Period of abstinence from alcohol prior to transplantation	
<6 months	7 (17)
6–12 months	7 (17)
1–5 years	17 (41.5)
More than 5 years	2 (4.9)
Blank	8 (19.5)

Thirty-three participants (80%) had a period of abstinence from alcohol before transplantation. This question was asked given the historic requirement of a 6 month “sobriety period” prior to transplant. The majority (41.5%) were had a 1–5 year period of abstinence prior to LT (Table 1). When asked about current alcohol consumption (AUDIT-C questionnaire), 88.6% of participants reported they never use alcohol, 5.7% of participants had alcohol 2–3 times a week, and 5.7% use alcohol drink monthly or less. When assessing participants' current intention to change their current alcohol consumption using the Stages of Change questionnaire, 62.9% of participants had been making an effort to change their alcohol use habits for more than 6 months (Maintenance Stage). 31.4% stated they were not doing anything to change their alcohol use habits and had no intention of doing so over the next 6 months (Pre-contemplation). These participants in the pre-contemplation stage also all indicated they were not currently using alcohol.

### Resources used before and after liver transplant

**Figure 1:**
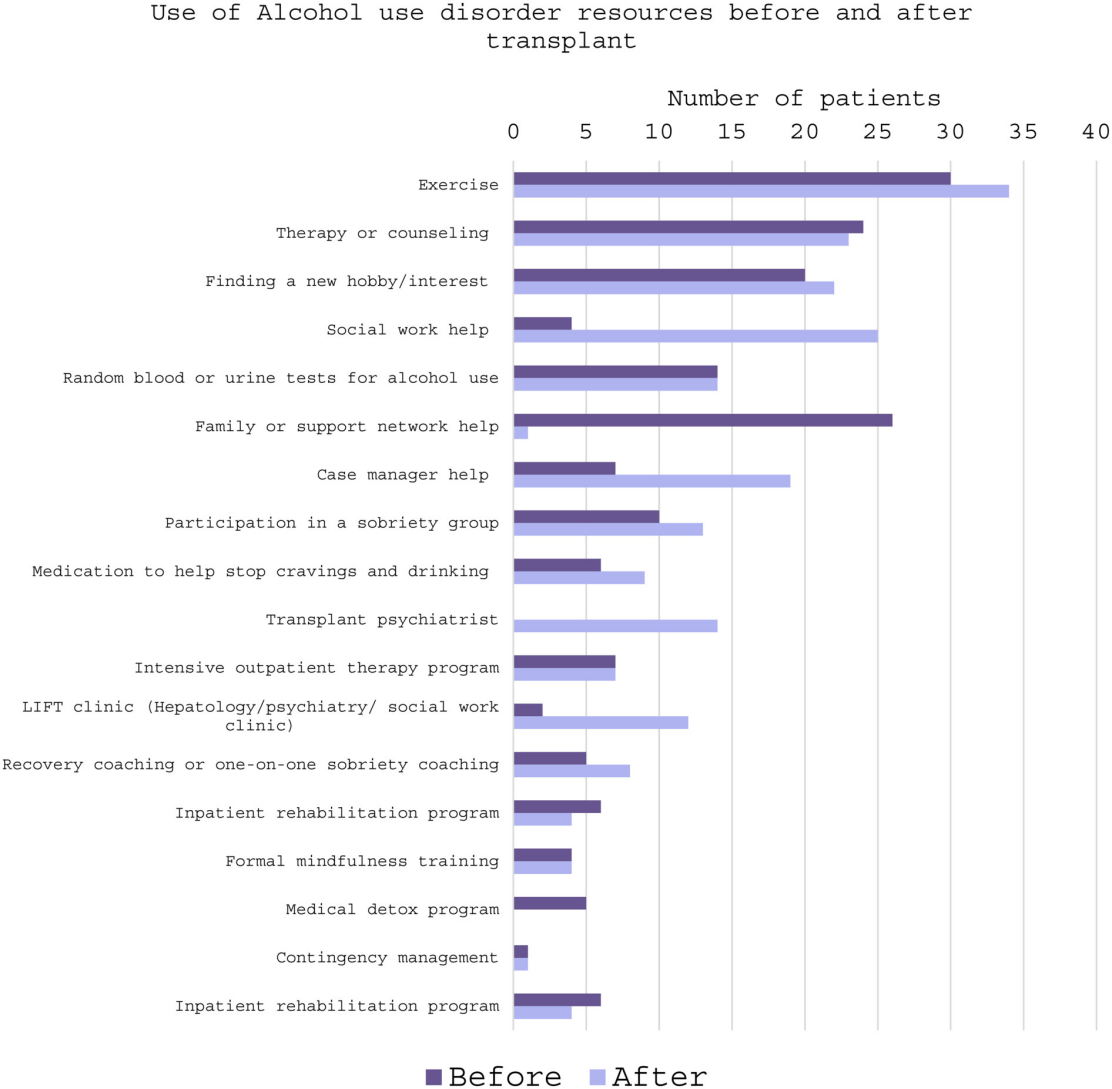
Frequency of use of alcohol use disorder management resources before and after liver transplant.

**Figure 2:**
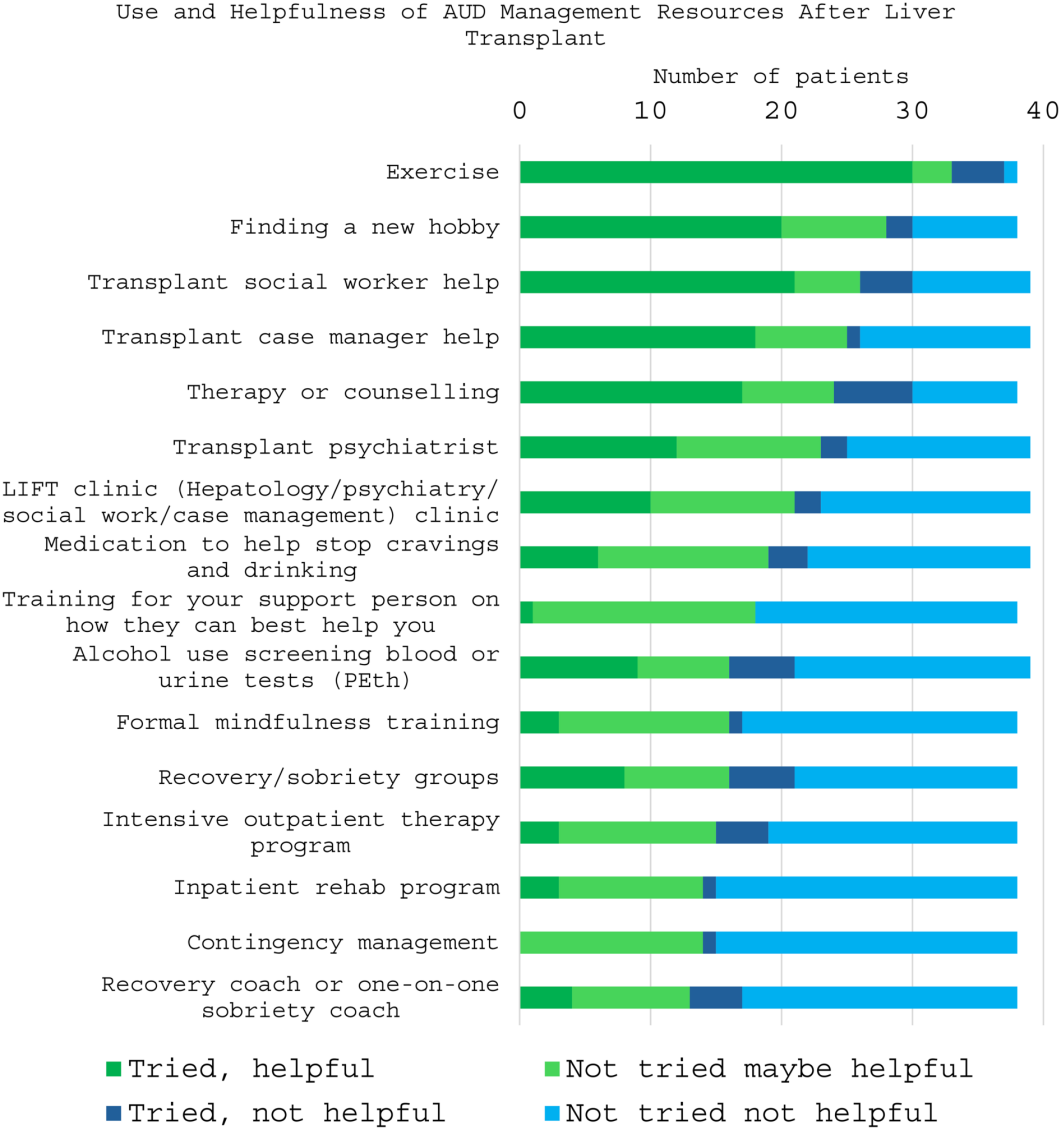
Perception of use and helpfulness of alcohol use disorder management resources after liver transplant. Patients were asked to identify if they had tried using the resource and if it was helpful vs. if they had not tried and felt it could be helpful or not. Resources with the more green proportion had been or were perceived to be the most helpful.

Regarding the most used resources to manage alcohol use before LT, exercise (73.1%) was the most utilized, followed by support from family or social network (63.4%), and therapy or counseling (58.5%). When asked about *potential* resources they might use after LT to avoid return to alcohol use, exercise (82.9%) social work assistance (61%), and therapy/counseling (56%) were the most used amongst the cohort (Figure 1). Similarly, when discussing the resources actually used after LT, participants described having tried and found helpful, exercise (73%), social work help (51.2%), and finding a new hobby (48.8%). Participants were also asked what resources they had not previously tried but still felt may be helpful. Participants indicated that training support persons on how to best help LT-patients (41.5%), contingency management (financial incentives for sobriety) (34.1%), medications to reduce cravings and alcohol intake (31.7%), and formal mindfulness training (31.7%), were resources that participants have not tried but thought might be beneficial (Figure 2).

When participants were specifically asked about the main difficulties to take medication for alcohol use disorder management, 37.5% of participants indicated high costs, 35% had concerns about medication side effects, 32.5% selected they were already taking many medications, and 25% were concerned about liver damage. Importantly, 32.5% indicated they did not believe there were additional barriers to medication for AUD.

Participants were asked an open-ended question about how social support may impact post-LT patients' ability to achieve their alcohol use goals. Participants listed continuous support through frequent contact with in-network transplant providers tracking their progress and transplant case management as the most helpful in terms of social support. Similarly, participants were asked an open-ended question about desired resources to add to their care to achieve alcohol use goals. Regular blood and urine alcohol screening tests, access to “out of network resources” such as a recovery coach or someone to contact when cravings present, immediate on-demand counseling, and a space to meet and share their stories with other patients facing the same diagnoses were desired.

### Challenges in AUD management

The majority of participants (89%) had a goal of complete sobriety or no use of alcohol. Similarly, 90.2% of participants indicated that the transplant center goal was complete sobriety. Responses to an open-ended question about the greatest challenge faced when trying to meet alcohol use goals included answers such as dealing with mental (depression, stress, work environment, habits); physical (pain, difficulty sleeping); and social (alcohol in social situations, family dynamics, expectations of others, care team lacking understanding of patient culture) challenges.

Participants were asked to select if they faced challenges across four different domains: social supports, resource availability, logistic challenges, and liver-transplant specific concerns. The most reported challenges were from the social support and resource availability domains, followed by logistic challenges, and finally, LT-specific challenges. 31.7% of participants reported no challenges in AUD care across all four domains (Table 2).

**Table 2:** Perceived challenges in alcohol use disorder management by liver transplant patients.

Challenges in AUD management	% yes
Social support challenges	
There is still alcohol in the place where I live	22.2
My family or friends do not support me in my alcohol use goals	11.1
My family or friends still use alcohol, and it is challenging for me	10.8
Other social support challenges	5.5
Availability of resources in my home community	
Difficulty finding a therapist	22.2
Difficulty enrolling in an intensive outpatient program	16.6
Difficulty finding an addiction counselor	16.6
Difficulty enrolling in an inpatient rehab program	13.8
Difficulty enrolling a detox program	13.8
Other resource limitations	11.1
Difficulty enrolling a sobriety group	2.7
Logistic challenges	
Programs or medications for alcohol use are too expensive	13.9
Transportation difficulties to get to treatment programs	11.1
Other logistic challenges	8.5
Insurance will not cover treatment programs or providers for alcohol use	8.3
Insurance will not cover medications for cravings or alcohol use	5.5
I cannot use/attend treatment programs or see providers and keep my job	2.7
Liver transplant-specific challenges	
I do not feel I can tell providers at the transplant center when I'm drinking or wanting to drink	8.3
Other liver transplant related challenges	8.3
Too many appointments to attend because of my liver transplant to be able to attend treatment for alcohol use	2.7
It is not clear what the providers at the transplant center want me to do	2.7
The transplant center expectations for my alcohol use do not align with my expectations	2.7
There is not enough support from the transplant clinic	2.7
I am back in the hospital often because of my liver transplant so I cannot regularly attend treatment for alcohol use	0
The transplant center expectations regarding alcohol are unreasonable	0

In the *social support* domain, the most reported challenge was the presence of alcohol at home (22.2%) followed by lack of support from family and friends in alcohol use goals (11.1%) (Table 2). Two participants reported “other” challenges, including limited work ability, boredom, and self-isolation. In the open-ended responses, around half of participants have not had any changes in their social support system after transplant, and the other half had a positive change in their support system. Regarding *resource availability*, 22.2% of participants reported having difficulty finding a therapist, followed by 16.6% with difficulty finding addiction counselors. 16.6% faced difficulty enrolling in intensive outpatient programs (Table 2). Four participants reported “other” challenges around availability of resources, including lack of financial help, and lack of advertisement of available opportunities for AUD treatment in their local community.

The most common *logistic challenge* participants felt might prevent LT patient from accessing care for AUD management after LT was the high cost of programs and medications for alcohol use disorder (13.9%). 11.1% identified transportation difficulties to treatment centers (Table 2). Three participants described “other” logistic challenges including lack of guidance on how to start treatment and time-management constraints when working or caring for others. Regarding *liver transplant -specific challenges*, 8.3% of participants were not able to tell providers when they were drinking or craving alcohol (Table 2). Three participants mentioned other LT-specific challenges including medication side effects, physical constraints related to transplant such as mobility difficulties, sleep difficulty and pain, comorbidities, and stigma associated with shame and failure when discussing alcohol use with transplant providers.

### Pre-LT limited abstinence subgroup

A total of 10 patients presented with limited abstinence prior to LT. Three were using alcohol until the transplant hospital admission and seven had <6 months of abstinence. All these patients reported abstinence after transplant and at the time of the survey. Similar to the entire cohort, these patients prioritized mental health (therapy or counseling) (*n* = 5) and exercise (*n* = 3) as one of the top three most helpful resources for AUD management. Interestingly, participation in a sobriety group (*n* = 3) was also selected by these participants as one of the most important resources which was not true in those with longer pre-LT abstinence.

### Post-LT non-abstinent subgroup

A small portion of the sample (4 participants - 9.8%) reported alcohol consumption at the time of survey completion. As a subgroup analysis, the survey responses were reviewed for these four participants. Prior to transplant, 2 had a period of abstinence of 6–12 months, one between 1 and 5 years, and one had more than 5 years of abstinence. Before LT, all participants had used exercise and family support, and 3 participants had tried therapy or counseling and finding a new hobby to not use alcohol, which was similar to participants reporting sobriety. After LT, the most helpful resource for all four participants was random urine and blood screening for alcohol use, which was different from the overall result of the cohort where the majority reported exercise to be the most beneficial. The next most selected resources were social work help and exercise (helpful for 3 of 4 participants), similar to the rest of the cohort. Three participants had seen a transplant psychiatrist or a therapist and found them helpful. Similarly, three participants used case manager help, finding a new hobby or the alcohol/hepatology combination clinic and found it helpful. This small cohort reported that recovery groups or recovery coaching was not helpful. Participants who did not use a recovery coach still did not think it would be helpful. Regarding challenges to achieve alcohol use goals, social support or logistic challenges were not the main concerns in this subgroup. Two participants reported challenges regarding availability of resources and ability to enroll in management programs, which was not a challenge identified for participants reporting sobriety. Two participants reported LT-specific challenges including that the transplant center goals did not align with their goals, and that they could not tell transplant providers when they were drinking, which was also was unique to these four participants. For the open-ended question, these four participants had heterogenous concerns about challenges with none of the four actively using alcohol reported the same challenges. One identified no challenges but still reported continued weekly alcohol use.

## Discussion

Previous studies have elucidated the providers' perception of barriers and use of resources for AUD in LT patients and patients with cirrhosis, but little is known about LT patients' perceptions (7, 12). To address this knowledge gap, we conducted a survey-based study of post-LT patients with a history of AUD. Participants identified the following challenges and barriers to reaching their alcohol use goals: the presence of alcohol at home, difficulty finding a therapist, and high cost of programs or medication for AUD. Participants identified exercise, family support, and therapy as beneficial to maintaining abstinence from alcohol in the pre-transplant setting. In the post-transplant setting, exercise, social work support and finding a new hobby were the most useful patient reported resources in their journey of AUD recovery (7, 12).

### Exercise

Exercise was identified as the most useful strategy by participants both before and after LT for AUD management. Previous studies have emphasized the positive effects of acute physical activity (short bouts of moderate intensity exercise) on alcohol consumption (13). Acutely, exercise has been shown to reduce alcohol cravings, mood disturbance, and anxiety, with larger magnitude effect on severe AUD compared to mild AUD (13). Similarly, patients performing a moderate level of physical activity presented with lower odds for excessive drinking than patients with low level physical activity, and patients reported decreased alcohol consumption for each increased exercising day (14). Exercise has also been proven to reduce symptoms of concomitant depression and anxiety in patients with AUD (15). In qualitative studies, exercise has been reported to reduce alcohol cravings and encourage healthier decision-making regarding alcohol intake (16). Our study similarly finds that LT recipients have found utility in exercise as a mechanism to achieve their AUD goals. While this was the most often selected facilitator of AUD recovery identified by survey participants, others did cite some of the limitations in AUD recovery were related to physical repercussions of LT. Future work should assess an exercise-based intervention that is appropriately adjusted for post-LT physical recovery stage.

### Family/social support

Family and social support play a crucial role in the successful treatment of alcohol use disorder. Low social support can be a predictor for relapse to alcohol use (17). In our study, half of the cohort reported that their support system had strengthened after liver transplant. Prior studies have indicated that a reliable social support network to help individuals cope with post LT challenges is essential for maintaining abstinence from alcohol after a liver transplant (4). In our cohort, several participants identified the presence of alcohol at home and social situations where alcohol played a significant role to be challenges they faced, which is consistent with findings in the literature (18). Targeted strategies, such as establishing alcohol-free environments with a sober community and educating support persons on the need to reduce exposure to alcohol may be strategies to help LT patients achieve their alcohol use goals.

### Professional resources: therapy and social work support

Psychotherapy interventions and social work support are critical for lasting change in AUD treatment, which was consistent with what this post-LT cohort reported (19, 20). In the pre-transplant setting, psychotherapy for AUD is associated with reduced incidence and progression of alcohol-related liver disease and lower rates of hepatic decompensation among patients with cirrhosis (20). While individual psychotherapy alone is not always sufficient to maintain abstinence, integration of psychotherapy with medical care and lifestyle changes is effective in reducing relapse risk (21, 22). Previous work has shown that the incorporation of a multidisciplinary team for post-LT and AUD management, including addiction specialist psychiatrists, social workers, and patient coordinators or case managers, reduced relapse in a small cohort of patients after liver transplant (23). In our study, participants did report challenges accessing AUD treatment resources and financial difficulties may limit pursuing adequate mental-health support including therapy. Strategies to help post-LT patients overcome barriers in accessing therapy could include: including a therapist in addition to a transplant social worker on the multidisciplinary liver-transplant team, providing financial and/or transportation support, and tele-health delivered psychotherapy (20–22).

### Leisure activities

Survey participants identified that finding a new hobby was a useful tool to manage AUD after LT. Several studies have described the role of hobbies in mitigating alcohol consumption in the general population with AUD. In one UK study measuring chronic stress, the frequent participation in activities such as art, cultural events, or visits to museums was associated with lower frequency of alcohol consumption and smoking (24). Similarly, in a qualitative study exploring the perception of alcohol reduction tactics in higher-risk drinkers, participants identified breaking old habits and taking up new hobbies as effective (25). We did not find studies specifically studying the impact of hobbies in post-liver transplant patients with AUD. Further research on the potential effectiveness of new hobbies and habits in alcohol consumption reduction in LT recipients is needed.

### Liver transplant-related challenges

For individuals with alcohol-related liver disease and a history of alcohol use disorder, liver transplant can present unique challenges in AUD recovery. These include managing side effects of immunosuppression medications, coping with physical limitations from the surgical recovery including mobility difficulties, addressing chronic pain and sleep disturbances as evidenced by responses from patients in this cohort. Specifically for transplant patients with AUD, psychological challenges of shame and stigma, can complicate discussions with healthcare providers (26). In some cases, patient care teams may lack understanding of cultural or family dynamics, which can limit the effectiveness of AUD treatment (5). These challenges highlight the need for comprehensive multimodal post-transplant care that not only addresses physical recovery but also supports the mental and emotional well-being of liver transplant recipients.

### Limitations

This study has some limitations. First, the low response rate to the survey, while seen in other non-transplant cohorts regarding alcohol use may limit the generalizability of the findings (27, 28). Notably, this study cohort was mainly composed of participants reporting no current alcohol use, which may be from a participation bias, and non-responding patients may be those with ongoing alcohol use (29). This limits the ability of our study to identify challenges for patients for patients with ongoing alcohol use. However, this also poses an opportunity to highlight the resources useful for patients with reported sustained abstinence. The single-center nature of our study limits the generalizability of our findings to broader populations of LT patients with AUD across other regions where there may be different resources available at transplant centers. In addition, self-reported data from patients is subject to recall bias, especially for the resources used prior to liver transplant and perception of their usefulness. The median time from transplant to survey completion was 1.7 years which could affect recall of resource utilization prior to transplant. Also, patients at different stages after LT and at different stages in their alcohol use recovery journey may require different resources for AUD treatment and may face different challenges. Finally, the lack of longitudinal follow-up does not allow to capture changes over time in patients’ perceptions or needs.

## Conclusion

Liver transplantation presents a unique set of challenges for patients with a history of AUD due to the complexity of both LT recovery and AUD recovery. Medication side effects, physical limitations, and emotional barriers including shame and stigma may hinder post-LT recovery, especially including AUD recovery. This cohort of post-liver transplant patients identified exercise and hobbies, social supports, and professional support through therapy and social work as the most useful resources to promote sustained AUD recovery after LT. Further work is needed to better understand nuanced challenges of patients with very limited or no pre-LT alcohol abstinence and those with ongoing alcohol use after LT.

## Data Availability

The raw data supporting the conclusions of this article will be made available by the authors, without undue reservation.
